# Validation of the German form of the Classroom Community Scale (CCS-D)

**DOI:** 10.3205/zma001737

**Published:** 2025-02-17

**Authors:** Harald Knof, Thomas Shiozawa

**Affiliations:** 1 Eberhard Karls University of Tuebingen, Faculty of Medicine, Department of Anatomy, Institute of Clinical Anatomy and Cell Analysis, Tuebingen, Germany

**Keywords:** medical education, interdisciplinary placement, factor analysis, validation, classroom community

## Abstract

**Background:**

An important prerequisite for collaborative learning is the integration of learners into a community. This supports individual learning processes and creates a common learning culture. The “sense of community” construct includes feelings of belonging and socio-emotional bonds with key elements including interdependence, trust, interactivity, and shared values. “Learning communities” in educational environments consist of two components: a sense of connectedness among members and shared learning expectations. The “Classroom Community Scale (CCS)” was developed to capture sense of community in collaborative learning environments. So far, this instrument is not available in German. Aim of this work is the translation and internal construct validation of a German form of the Classroom Community Scale (CCS-D).

**Methods:**

The questionnaire was administered to N=334 first semester students in the programs of human medicine, dentistry, and molecular medicine at the Eberhard Karls University of Tuebingen, Germany. Descriptive analysis, as well as a confirmatory and principal component analysis were performed.

**Results:**

Cronbach’s α=.87 could be recorded for the overall questionnaire, with reliabilities of α=.85 for the subscale Connectedness and α=.76 for the subscale Learning. In confirmatory factor analysis, the model achieves moderate (CFI=.85; TLI=.83) to acceptable (χ^2^ [169, n=334]=455.368, *p*<.000; χ^2^/df=2.694; RMSEA=.071; SRMR=.0605) model fit.

**Discussion:**

The reliability of the CCS-D demonstrates results similar to those found in existing literature. The two-factor structure of the model could be confirmed, with moderate to acceptable model-fit.

Therefore, the CCS-D is a usable instrument to measure sense of community in learning environments.

## Zusammenfassung

**Hintergrund:**

Eine wichtige Voraussetzung für kollaboratives Lernen ist die Integration der Lernenden in eine Gemeinschaft. Dies unterstützt individuelle Lernprozesse und schafft eine gemeinsame Lernkultur. Das Konstrukt des „sense of community“ („Gemeinschaftssinn“ oder „Gemeinschaftsgefühl“) umfasst Zugehörigkeitsgefühle und sozio-emotionale Bindungen mit Schlüsselelementen wie Interdependenz, Vertrauen, Interaktivität und gemeinsamen Werten. „Lerngemeinschaften“ in Bildungsumgebungen bestehen aus zwei Komponenten: einem Gefühl der Verbundenheit unter den Mitgliedern und gemeinsamen Lernerwartungen. Die „Classroom Community Scale (CCS)“ wurde entwickelt, um das um das Konstrukt „Gemeinschaftsgefühl“ in kollaborativen Lernumgebungen zu erfassen. Bisher ist dieses Instrument nicht auf Deutsch verfügbar. Ziel dieser Arbeit ist die Übersetzung und Validierung einer deutschen Version der Classroom Community Scale (CCS-D).

**Methoden:**

Der Fragebogen wurde N=334 Studierenden im ersten Semester der Studiengänge Humanmedizin, Zahnmedizin und Molekulare Medizin an der Eberhard Karls Universität Tübingen, Deutschland, vorgelegt. Es wurden deskriptive Analysen sowie eine konfirmatorische Faktoren- und eine Hauptkomponentenanalyse durchgeführt.

**Ergebnisse:**

Für den gesamten Fragebogen konnte Cronbachs α=.87 verzeichnet werden, mit Reliabilitäten von α=.85 für die Subskala *Verbundenheit* und α=.76 für die Subskala *Lernen*. In der konfirmatorischen Faktorenanalyse erreicht das Modell einen moderaten (CFI=.85; TLI=.83) bis akzeptablen (χ^2^ [169, n=334]=455.368, p<.000; χ^2^/df=2.694; RMSEA=.071; SRMR=.0605) Modell-Fit.

**Diskussion:**

Die Reliabilität des CCS-D zeigt vergleichbare Ergebnisse wie in der bestehenden Literatur. Die Zwei-Faktoren-Struktur des Modells konnte bestätigt werden, mit moderatem bis akzeptablem Modell-Fit. Daher ist der CCS-D ein nutzbares Instrument zur Messung des Gemeinschaftsgefühls in Lernumgebungen.

## 1. Introduction

“Research has shown, that learning involves knowledge acquisition through cognitive processing from individual thought processes as well as from being part of a society” [1].

To address the social part of learning, collaborative learning and team-based approaches are increasingly implemented in medical education and can take many different forms: face-to-face, digitally, synchronously, or asynchronously [2], [3], [4]. Due to collaborative learning students experience effective teamwork behaviors, communication competencies and a feeling of responsibility for their own performance as well as team performance, a necessary skill in future professional life, that can impact on the quality of health care [5], [6]. However, students still face several challenges in collaborative learning. Group work can be carried out with unequal individual participation, communication can be ineffective and dealing with group members can be difficult [7], [8], [9]. This also makes it difficult for the medical education community to consistently achieve better results through the use of collaborative learning methods and strategies [10]. A parameter to foster collaborative learning is the feeling of how the students feel integrated into their peer group. A psychological construct to describe this phenomenon is the so called “sense of community” [11]. It has been shown that students’ collaborative learning is significantly correlated with sense of community [12].

### 1.1. Sense of community 

Although there is extensive literature, there is no generally accepted definition of the term “sense of community” [11], [13], [14], [15]. Some authors define sense of community as a feeling of membership and shared socio-emotional connections [16]. Some other authors emphasize the perception of similarity and interdependence with others [17].

A widely used definition occurs from McMillan and Chavis: “Sense of community is a feeling that members have of belonging, a feeling that members matter to one another and to the group, and a shared faith that members’ needs will be met through their commitment to be together” [11]. Sense of community refers to variables that go beyond individual behavior and individual relationships [18]. The most essential elements of sense of community are spirit, trust, mutual interdependence among members, interactivity, shared values and goals [13], [19]. It should be noted that the dimensions of community and so the sense of community differ depending on the setting [18], [20], [21]. A wide variety of settings, like neighborhoods, workplaces or virtual spaces, have already formed the basis for discussions and work on sense of community [22]. Learning environments represent a special setting, and have to be considered separately [23].

### 1.2. Classroom community 

Classrooms represent a special form of a psychological community. The pivotal factors are: The setting is the learning environment; the purpose of community is learning; the community is timebound, e.g. to the duration of the course or program in which the members are enrolled [24]. According to Rovai, and based on the theoretical framework described above, the characteristics of a sense of classroom community include feelings of connectedness, cohesion, spirit, trust, interaction, and shared educational goals, in this case learning [13], [24], [25]. Adapted on the special setting of classroom and educational environments, classroom community comprises two components: feelings of connectedness among community members and commonality of learning expectations [13].

Connectedness represents the recognition of belonging to a community. Feelings of friendship and cohesion develop among the learners. Once individuals are accepted as part of a learning community, they develop a sense of security and trust, while trust has to be seen as the feeling that the community can be trusted and feedback will be positive and immediate [13], [26]. This feeling of trust is accompanied by the willingness of community members to speak openly, which is important, because with this trust, members are more likely to expose learning gaps, and feel that other members of the community will respond in a supportive manner [13], [24].

*Learning* is the feeling that knowledge and meaning are being built actively within the community. In doing so, the community promotes the acquisition of knowledge and understanding, and the learning needs of its members are met. Members not only have to identify with the group, but also accept the values and goals of the group [13]. Learning is that goal and represents an indispensable part of classroom community [23].

Based on this work “classroom community” can be defined as a social community of learners who share knowledge, values, and goals [13]. It is known that a distinctive sense of classroom community is associated with students’ well-being and learning progress [12], [27], [28], [29], [30]. Sense of classroom community predicts academic outcomes like effort and is positively related to student success and exam performance, both in in face-to-face and online classes [21], [31], [32], [33], [34], [35]. The general trend of online learning formats and blended learning was exacerbated by the COVID 19 pandemic. Here in particular, it is important to pay more attention to the sense of community, since this is more difficult to achieve in blended learning formats than face-to-face [36]. Educational environments that promote sense of classroom community lead students to a feeling of safety, value and respect while foster learning and engagement and therefore supports and challenges, but most importantly enriches students in their intellectual experience [37], [38]. Based on that, the potential for learning with others is greater than learning alone [39]. On the other hand, students with lower sense of classroom community are more likely to become dropouts [40]. Educators have to aim on building and sustaining strong feelings of community, as they may prevent these dropouts by increasing support, collaboration, commitment to group goals, and the satisfaction with academic efforts [41], [42].

As described above, collaborative learning and team-based approaches are increasingly utilized in medical education with multiple intentions, but challenges such as unequal participation and ineffective communication persist. A crucial part is the sense of classroom community, persisting of the components *connectedness among members* and* shared educational objectives*. The recognition of the importance of fostering a sense of community within learning environments lead to the development of the Classroom Community Scale (CCS), an instrument for measuring the sense of community in collaborative educational environments [43]. This empowers educators to address the challenges and enables better exploration of how collaborative educational environments can be best designed and implemented [43].

### 1.3. Classroom Community Scale

The Classroom Community Scale contains 20 items in two subscales – Connectedness and Learning – and is shown in table 1 (Tab. 1). 

The subscale *connectedness* consists of 10 items (odd numbers) related to feelings of connectedness. The subscale *learning *contains 10 items (even numbers) related to feelings about the use of interaction within the learning community. Participants rate each item on a 5-step Likert scale from *strongly disagree *to *strongly agree*. To get the total Classroom Community Scale score (maximum score=80), the values of all 20 items are added together. Each subscale can reach a maximum of 40. Higher scores on the total Classroom Community Scale indicate a stronger sense of classroom community while lower scores indicate a less strong sense of classroom community [43].

The Classroom Community Scale instrument was employed in various research its original form to assess the sense of community in face-to-face, blended, and virtual learning environments sometimes supplemented by additional validations [44], [45], [46], [47], [48]. Additionally, translated versions of the instrument in Italian [49] and Persian [50] have been validated and utilized in similar settings. Furthermore, a short form of the original instrument (CCS-SF) was developed [51].

To our knowledge, in medical education literature there is no such instrument available for measuring sense of community in collaborative learning environments in German-speaking countries so far. Aim of this article is to describe translation process of the Classroom Community Scale into German and to demonstrate reliability and factor structure of the German Version of classroom community scale (CCS-D).

## 2. Methods

### 2.1. Translation process

After receiving permission for translation from the developer of the original CCS-instrument translation was performed according to international guidelines using forward-backward-translation [52]. The CCS-instrument was first translated from English to German by a native speaking medical student in cooperation with the authors to maintain underlying concepts of the questionnaire. This reconciled German version was then translated back to English by another native speaker with medical background. During the translation process, questions arose about the exact definition and translation of individual items, as for example when talking about the exact translation of the word “isolated” in item 9 (see table 1 (Tab. 1)). Also, in item 8 there is a difference between “sharing gaps” and “exposing gaps” (see table 1 (Tab. 1)). These questions were solved with the authors and German wording was adapted accordingly.

### 2.2. Data collection

In the finals weeks of winter semester 21/22 and summer semester 2022, a total of *N*=344 first semester students at the Faculty of Medicine Tuebingen were surveyed using a paper-based questionnaire. At this point, the participants had completed all courses of the first semester of their medical study program. Students were invited to participate by the first author in person. For their participation, the students received an expense allowance of €5, funded by the Faculty of Medicine Tuebingen. Written informed consent was obtained from all participants prior to the study. They were informed about the study, chances, risks, rights, obligations, and the voluntariness of the study. Data were collected in pseudonymized form. All participants also agreed to the publication of the data in anonymized form. Students could revoke their consent without incurring any disadvantage. Ethical approval for this study was obtained from the ethics committee at Eberhard Karls University Tuebingen with letter no. 086/2022BO2.

### 2.3. Data analysis

As in the original CCS instrument for items 1, 2, 3, 6, 7, 11, 13, 15, 16, and 19 the scoring scale was: *strongly agree*=4, *agree*=3, *neutral*=2, *disagree*=1, *strongly disagree*=0; for items 4, 5, 8, 9, 10, 12, 14, 17, 18, and 20 the scoring scale was: *strongly agree*=0, *agree*=1, *neutral*=2, *disagree*=3, *strongly disagree*=4 [43]. For further analysis these item-scores must be inverted. These items are labeled with an “i” in tables and figures of this paper. 

Descriptive statistical analysis was carried out to present the sample and to determine the scale and item characteristics of the CCS-D. 

In line with the original publication by Rovai (2002) and before mentioned studies by Perrucci et al. (2022), Abdeldayem et al. (2020), Ahmady et al. (2018), Hur et al. (2013), Zhang et al. (2011), and Barnard-Brak & Shiu (2010), Cronbach’s alpha was employed to assess the internal consistency characteristics of the subscales as well as the consistency of the whole questionnaire. Values equal to or higher than 0.70 are deemed satisfactory, although it has been proposed that values reaching 0.80 are the minimum acceptable standard [53].

Construct validity of the 2-factor structure of the German version of the CCS instrument was assessed through Confirmatory Factor Analysis (CFA). The examination of external validity constitutes a central focus for prospective research.

First, the observed variables (10 *connectedness* items, 10 learning items) were entered into a CFA model. Then, the latent variables *(connectedness, learning)* were added to the model. Maximum likelihood fit was chosen because its conditions were met, as there were both a large sample and the data used continuous levels of measurement. Typically, a 5-point rating scale is considered an ordinal measure. The maximum likelihood adjustment can be applied if there are at least five rating points in each latent variable and at least three observed variables [54].

To assess the fit of the 2-factor-model, the following indicators were considered, based on international guidelines [55], [56], [57] and in order to compare CCS-D with previous validations [44], [45], [49], [50]: Normed Chi-square goodness-of-fit statistic, with a threshold for acceptability at χ^2^/df<3 [58]. Comparative Fit Index (CFI) and Tucker–Lewis Index (TLI), where good model fit is indicated at .95 (Hu & Bentler, 1999). In literature, previous research values for the Root Mean Square Error of Approximation (RMSEA) ranging from .05 to .08 were considered acceptable [59], [60], [61]. However, the most recent threshold for the RMSEA value is set at values <.06 [62]. The Standardized Root Mean Square Residual (SRMR) was also used, where values less than .08 are considered acceptable [62]. 

In conjunction with CFA, Principal Component Analysis (PCA) was employed to assess the model's robustness, emphasizing its data-driven nature, in order to explore the underlying factor structure and potential patterns in the data that might not have been captured by the pre-established assumptions of the CFA [63], [64], [65]. For this purpose, PCA was performed using varimax rotation with Kaiser normalization, only items with factor loading ≥|.30| on one or both dimensions were selected [58], [66].

For collecting questionnaire responses, extraction, and analyses SPSS Statistics 29 (IBM Corp., Armonk, NY) was used. CFA was done with IBM SPSS AMOS 28 (IBM Corp., Armonk, NY).

## 3. Results

### 3.1. Sample

A total of *N*=344 first-semester medical students at the Faculty of Medicine Tuebingen were surveyed using a paper-based questionnaire. With a total admission of 210 students per semester a maximum of 420 participants could have been included; this represents a response rate of 81.9%. To meet the conditions for use of AMOS in further analysis, all participants with any missing data for items of CCS-D were excluded. This resulted in 334 respondents.

Study participants were 69.2% female, corresponding to the gender distribution of the admission. The age ranged from 18 to 35 years; the mean age was 20.73 years (SD 2.736). Most participants were medical students (96.7%) followed by dentistry students (3.0%) and students of molecular medicine (0.3%).

### 3.2. Scale characteristics and internal consistency

Descriptive statistics for the individual items (see table 2 (Tab. 2)) as well as the total Classroom Community Scale and for each subscale are shown below (see table 3 (Tab. 3)). 

The total Classroom Community Scale (Cronbach’s alpha=.87) and the subscale *connectedness* (alpha=.85) showed good internal consistency. The subscale *learning* is reported with a Cronbach’s alpha=.76, indicating acceptable internal consistency.

### 3.3. Confirmatory factor analysis

**Figure 1:**
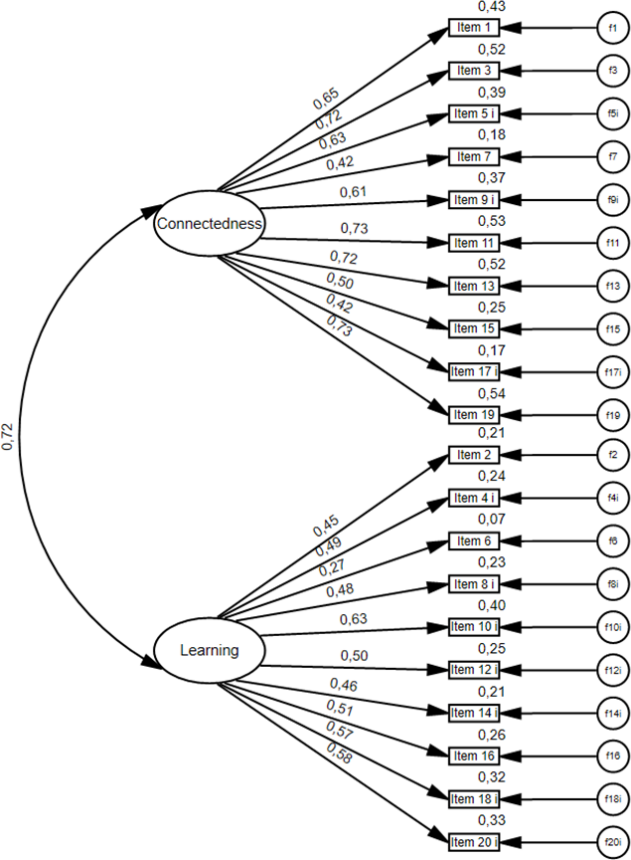
CFA model for the two factors of the German Form of Classroom Community Scale (CCS-D)

The CFA-Model for the 2-factor-model is displayed in figure 1 (Fig. 1).

The 2-factor-model showed an acceptable model fit regarding the normed Chi-squared (χ^2^ [169, n=334]=455.368, *p*<.000; χ^2^/df=2.694). The RMSEA (.071) and SRMR (.0605) showed an acceptable fit. The CFI (.85) and TLI (.83) indicating a moderate model fit. Standardized regression weights (factor loadings) are all >.4 except item 6 (“I feel that I receive timely feedback”).

### 3.4. Principal component analysis

The Kaiser-Meyer-Olkin measure was .881, representing a relatively good factor analysis. The Bartlett’s test of sphericity was significant (*approx. χ^2^*=2056.322; *p*<.001). The analyzed data therefore does not result in an identity matrix and is suitable for factor analysis. Only factors with eigenvalues ≥1 were considered [67], [68]. Examination of Kaiser’s criteria and the scree-plot lead to retain two factors with eigenvalues >1 which accounted for 39.5 % of the total variance. In the varimax-rotated two-factor model (see table 4 (Tab. 4)), most items only load on one of the two factors, except item 9 (“I feel isolated in this course”) and item 16 (“I feel that I am given ample opportunities to learn”). The underlying structure of the partitioning of the CCS into c*onnectedness* and* learning* can be verified, as the odd items are highly loading on factor 1, the even items on factor 2. Only item 14 (“I feel that other students do not help me learn“) loads on factor 1, even if it is actually a learning item.

## 4. Discussion

The CCS-D shows good reliability and replicates the factor structure of the original version in the CFA. The factor structure was also almost confirmed in the PCA performed. Values of internal consistency are quite similar compared to the original version as well as to other studies and translated versions (see table 5 (Tab. 5)). When considering the model-fit indices, the CCS-D performs better in some cases and worse in others. It should be noted that with little available literature on CFA of the CCS questionnaire, not all model fits are always reported (see table 6 (Tab. 6)).

Although the factor structure was replicated, a high correlation was found between the individual subscales *connectedness* and *learning*, suggesting that these subscales may not represent different dimensions of classroom community and influence each other. A likewise high correlation between the subscales has already been reported in the literature [44].

Also indicative of this are the cross-loadings of items 9 and 16, as well as the loading of item 14 on the subscale *connectedness* in PCA. These cross-loadings have already been described in the literature [49], [51], [69]. Some participants may have interpreted Item 9 (“I feel isolated in this course”) to mean that they did not feel included in the lesson by the teacher. This interpretation considers this item to be part of the learning dimension, in which the instructor plays a central role [49]. It is known, that a lecturer’s personality or disposition can influence students’ sense of social presence and class community [70], [71]. As described above, during the translation process, there were questions about the exact translation of the word “isolated”. The choice was less of a literal translation in the sense of “isolation” and more in the direction of “loneliness”, which can refer more to collaboration, as in the case of the meaning of the word “lonely”, less to relationships, as in the case of meaning of the word “alone”. A similar interpretation-shift maybe occurs for item 16 (“I feel that I am given ample opportunities to learn”), in which the possible interpretation focuses on learning opportunities in the presence of fellow students, and thus the connectedness component is more likely to be addressed [71]. It is plausible with Item 14 (“I feel that other students do not help me learn”), given its wording and content, that the interpretation of the item focuses on the quality of the relationship with their classmates, which is seen as the reason for the lack of help with learning. This brings the feeling of connectedness into focus, even though the item was originally designed as part of learning [49]. Thus, students’ general interest in developing a sense of community may be influenced by students’ expectations of how they will interact with fellow students in future courses and/or in their careers [72]. Item 9 and Item 14 were excluded in the process of creating the CCS-SF [51]. It should be considered whether a similar procedure can be used in case of a possible creation of a German-language CCS-D-SF. Inconsistencies between the current study and previous research may possibly be attributed to differences in sampling, methodology, and program/course distinctions.

There are some limitations to this work that should be considered. Due to the fact, that a part of data collection occurred in Winter 21/22, the COVID 19 pandemic could have impacted students’ sense of connectedness und collaborative learning [73], [74]. A homogenization of student communities can be observed, driven by similar and shared social and personal challenges, including uncertainties and the transition to online education, leading to a limitation of social contacts. Normally, students in the first semester usually focus on building personal relationships and building up a professional network relevant to their studies [75]. Social integration and personal interaction were more difficult through the pandemic, as contact with fellow students was mostly only possible digitally [76], [77]. In contrast to the present study, in which sense of community was measured over an entire academic program, most previous research using the CCS has focused on single courses or classroom sessions [78]. 

## 5. Conclusion

In this study the translation process and confirmatory factor analysis of the Classroom Community Scale (CCS) instrument into German Form (CCS-D) was presented. 

The reliability of the CCS-D can be compared with literature and yields similarly good results. In CFA, the two-factor structure of the model could be confirmed. The moderate to acceptable model-fit is comparable to existing values in previous research. The factor structure was also observed in the principal component analysis performed.

Therefore, the CCS-D is a usable instrument to measure sense of community in learning environments. Future research should focus on using this questionnaire in German-speaking countries. Further development can be carried out if larger amounts of data from different learning environments and course formats are available.

## Authors’ ORCIDs

- Harald Knof: [0000-0002-0942-653X]
- Thomas Shiozawa: [0000-0002-7112-1016]

## Conference presentation

Excerpts from this manuscript were presented at the Annual Conference of the DACH Association for Medical Education (GMA) in Osnabrück 2023 [79].

## Acknowledgements

The authors would like to thank Prof Alfred P. Rovai from Regent University (Virginia Beach, VA, USA) for the permission to use and translate his CCS instrument. The authors would like to thank Charlien Wolf and Lena Riha who performed the back-to-back-translation of the questionnaire. The authors would like to thank Prof. Dr. Peter Martus for statistical consulting. The authors are grateful to students at the Faculty of Medicine Tuebingen for participating in this study.

We acknowledge the support from the Open Access Publication Fund of the University of Tübingen.

## Competing interests

The authors report no conflicts of interest. The authors alone are responsible for the content and writing of this article. The authors declare that the research was conducted in the absence of any commercial or financial relationships that could be construed as a potential conflict of interest.

## Figures and Tables

**Table 1:**
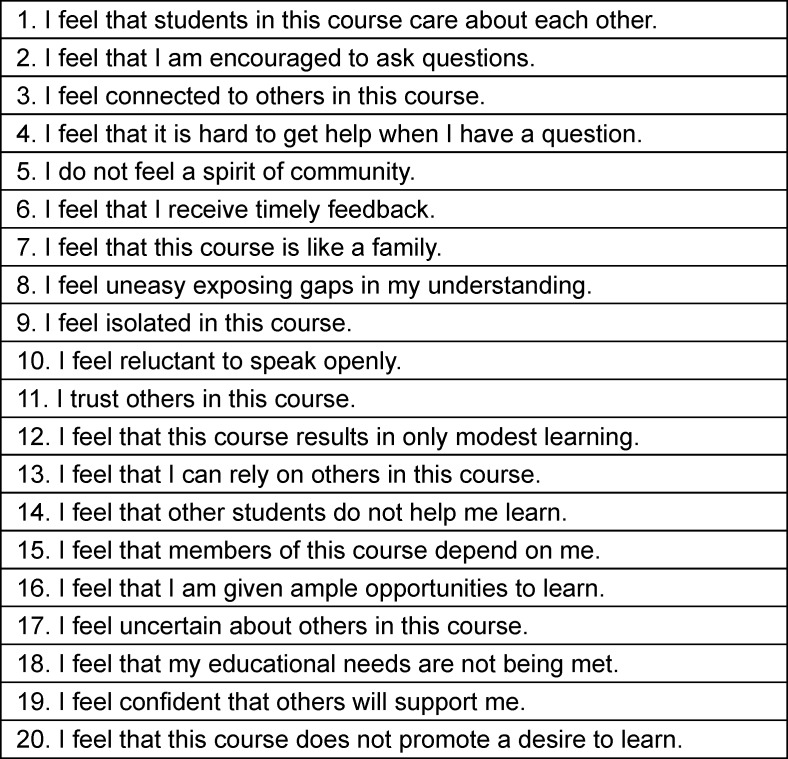
Classroom Community Scale by Rovai 2002

**Table 2:**
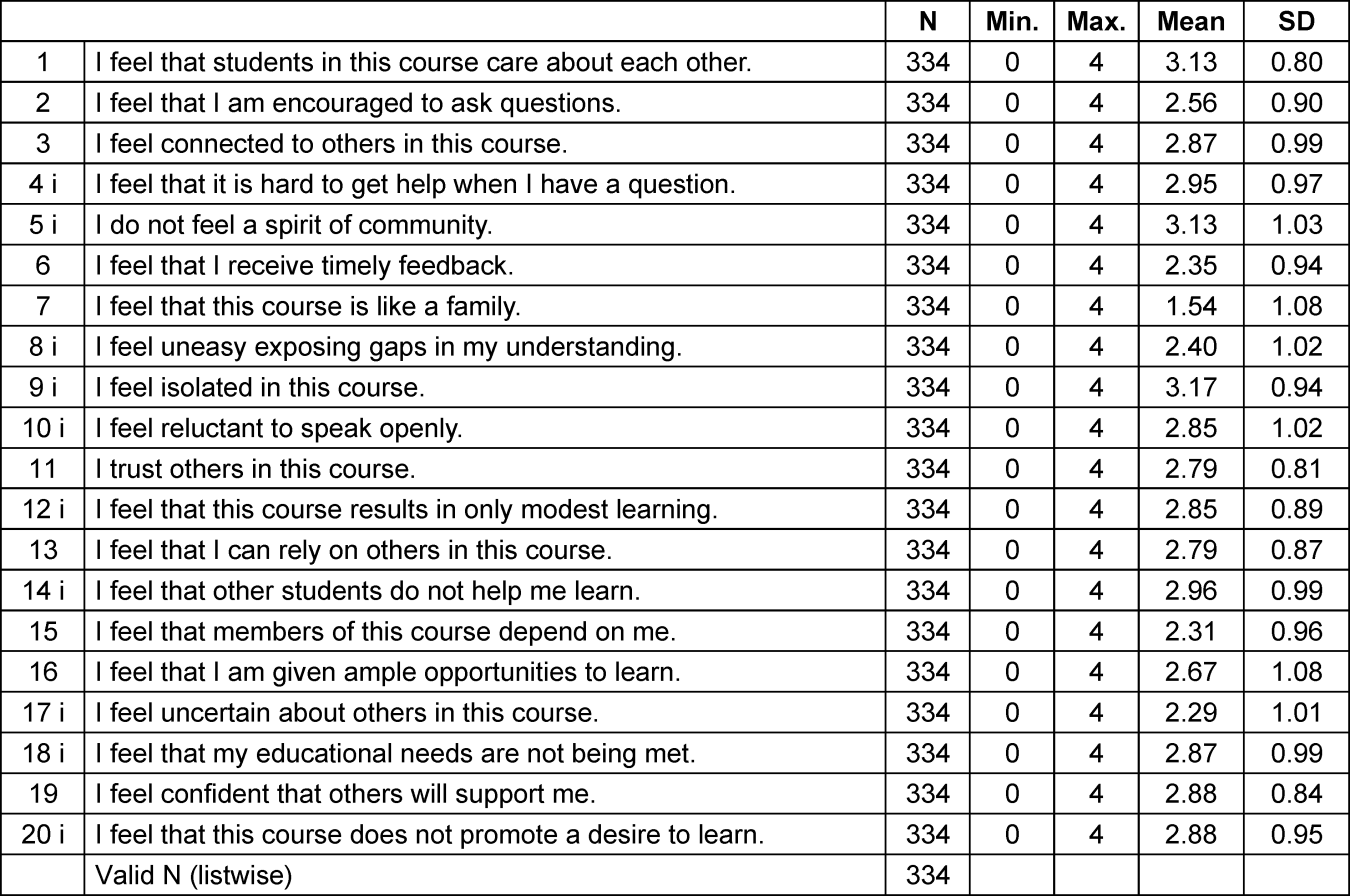
Descriptive statistics of individual items of CCS-D

**Table 3:**
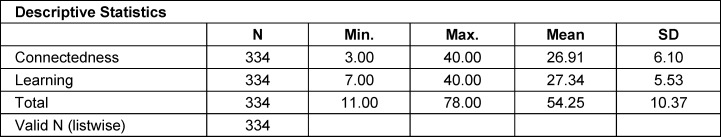
Descriptive statistics of subscales of CCS-D

**Table 4:**
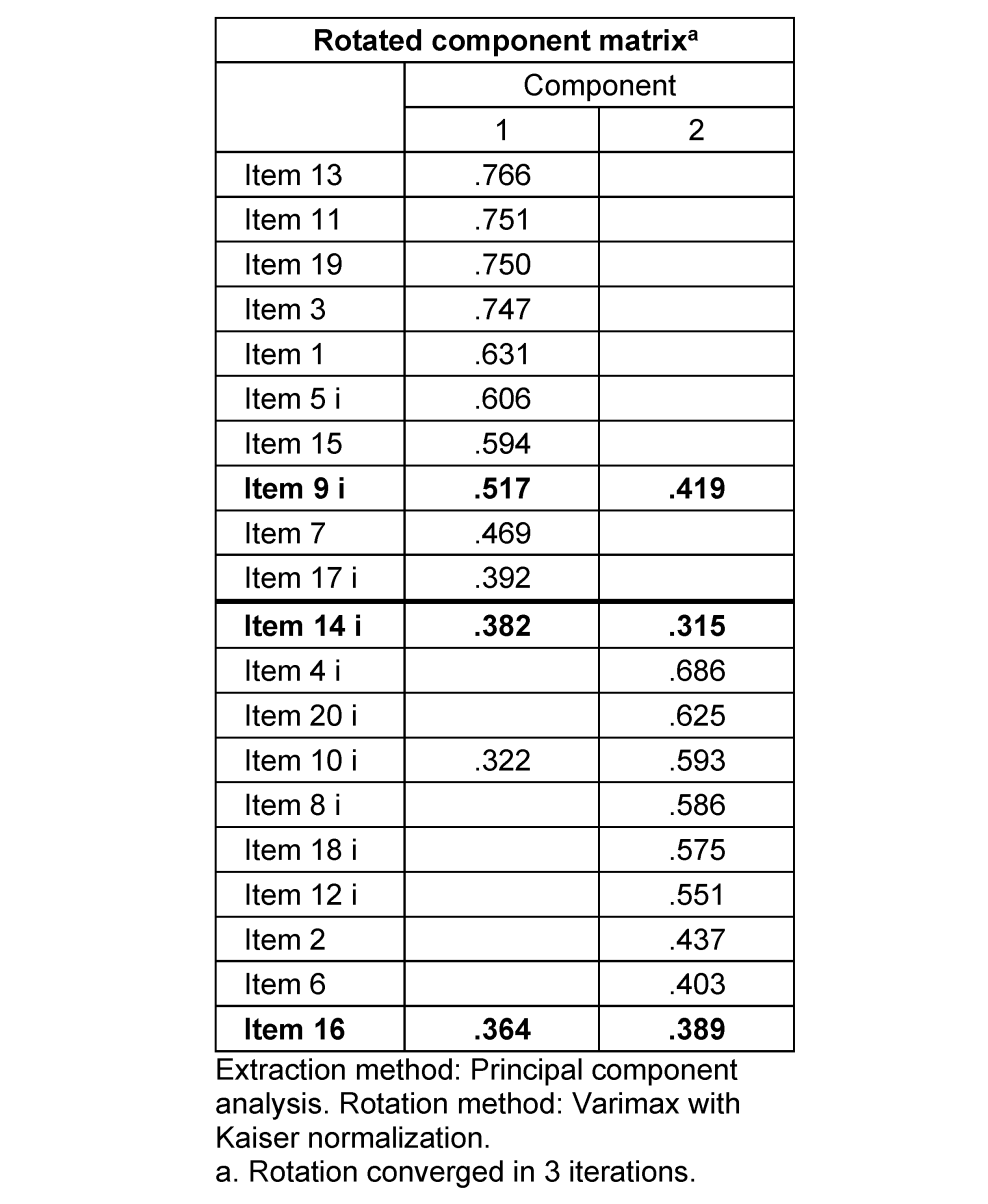
Varimax rotation PCA

**Table 5:**
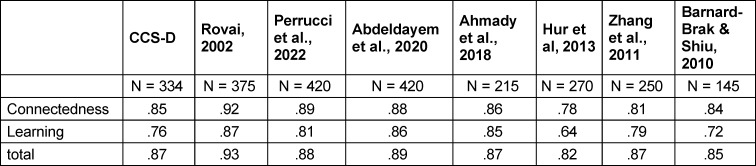
Internal consistency (Cronbach’s alpha) in comparison to other studies

**Table 6:**
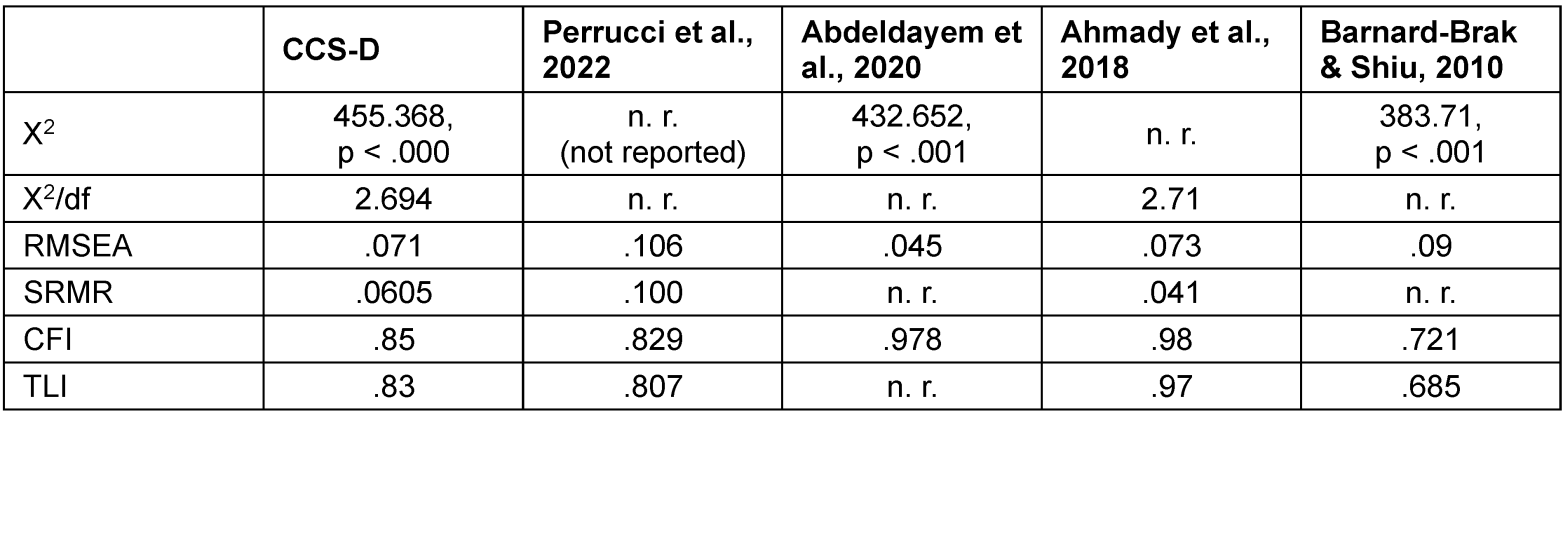
Model fit indices in comparison to other studies
